# Sperm-fluid-cell interplays in the bovine oviduct: glycosaminoglycans modulate sperm binding to the isthmic reservoir

**DOI:** 10.1038/s41598-023-37469-3

**Published:** 2023-06-26

**Authors:** Coline Mahé, Thanya Pranomphon, Karine Reynaud, Ludivine Laffont, Thierry Meylheuc, Jennifer Schoen, Pascal Mermillod, Marie Saint-Dizier

**Affiliations:** 1 CNRS, IFCE, INRAE, Université de Tours, PRC, 37380 Nouzilly, France; 2 School of Biotechnology, Embryo Technology and Stem Cell Research Center, Suranaree University of Technology, Nakhon Ratchasima, Thailand; 3 INRAE, Pathologie Végétale, 84143 Avignon, France; 4 Department of Reproduction Biology, Leibniz Institute for Zoo and Wildlife Research (IZW), Berlin, Germany; 5 Faculty of Sciences and Techniques, Tours University, Tours, France

**Keywords:** Cell biology, Molecular biology

## Abstract

When entering the oviduct for fertilisation, spermatozoa come into contact with the oviduct fluid (OF) and can bind to luminal epithelial cells in the isthmus to form a sperm reservoir. The objective of this study was to examine how the OF modulates sperm adhesion to the oviduct reservoir using an in vitro model of oviduct epithelial spheroids (OES). Bovine oviducts from a local slaughterhouse were used to collect OF and isthmic fragments for the in vitro incubation of OES. Compared to a non-capacitating control medium, the pre-ovulatory OF significantly decreased by 80–90% the density of spermatozoa bound to OES without affecting sperm motility, membrane integrity, or sperm-cilia interactions. This effect on sperm binding was reproduced with (1) OF from different cycle stages and anatomical regions of the oviduct; (2) OF fractions of more than 3 kDa; (3) modified OF in which proteins were denatured or digested and (4) heparan sulphate but not hyaluronic acid, two glycosaminoglycans present in the OF. In conclusion, the OF significantly decreased the number of spermatozoa that bind to oviduct epithelial cells without affecting sperm motility and this effect was due to macromolecules, including heparan sulphate.

## Introduction

Successful fertilisation in mammals relies on the well-timed meeting of spermatozoa and an oocyte in the oviduct. When entering the oviduct, spermatozoa come into contact with the oviduct fluid (OF) and some firmly binds to oviduct epithelial cells in the first part of the oviduct, called the isthmus, to form a sperm reservoir^1,2^.

The accumulation of sperm within the reservoir is believed to lengthen the life span of sperm in case of desynchrony between mating and ovulation, and promote fertilisation after sperm release^3–5^. Direct observations in the oviduct of mice have suggested that spermatozoa not only bind to isthmic epithelial cells but detach and reattach several times as they ascend toward the oocyte, displaying dynamic sperm-fluid-cell interplays prior to conception^6^. However, the ways in which the OF components regulate sperm binding to the oviduct epithelium remain unclear.

The OF is a complex biological fluid containing proteins, glycosaminoglycans, metabolites, hormones, and lipids that are highly regulated across the estrous cycle of cows^7^. Previous data from our lab identified 245 proteins in bovine OF that interacted with spermatozoa^8^ including those previously proposed as sperm receptors in the isthmic reservoir, i.e., annexins A1, A2, A4, A5, and heat shock proteins 60 and A5, among others^4,9,10^. Proteins in the OF are then expected to compete with the same proteins located on the oviduct epithelium and decrease the binding of sperm to epithelial cells.

In addition, hyaluronic acid (HA) is a high molecular weight non-sulphated glycosaminoglycan which is abundant in the OF^11,12^. It has previously been shown that HA-binding proteins are present at the surface of bovine oviduct epithelial cells (OEC)^11^ and on bull spermatozoa^13^. Motile bull spermatozoa have also been shown to be capable of binding to HA in vitro^14,15^. Therefore, HA is a good candidate for regulating sperm binding to the isthmic reservoir in cattle. Moreover, the sulphated glycosaminoglycan heparan sulphate (HS), which is also abundant in bovine OF^12^, has been shown to bind with a high affinity to the binder of sperm proteins (BSPs), a group of sperm surface proteins involved in sperm binding to the oviduct reservoir^16^. Heparin, which is chemically similar to HS but not reported in the OF, was previously found to inhibit bull sperm binding to OEC monolayers^16^ and induce the release of bound spermatozoa from cells within minutes in vitro^5,17^.

Therefore, we hypothesised that glycosaminoglycans and proteins in the OF interact with spermatozoa and decrease their ability to bind to the oviduct epithelium.

Numerous in vitro studies have assessed sperm interactions with OEC in chemically defined culture media^5,18–20^ and some others focused on the effect of OF on sperm physiology^21,22^. However, data on the effect of OF on sperm-OEC interactions are currently lacking. Moreover, most studies on sperm-cell interactions have used oviduct epithelial explants presenting a great heterogeneity in surface binding^18,19,23^, or epithelial cell monolayers which were rapidly losing their cilia^5,20,24^. The objective of this study was thus to evaluate the impact of OF and its components on sperm binding to the oviduct reservoir using a new, well-standardised and differentiated in vitro model of isthmic epithelial cell spheroids.

## Results

### Characterisation of oviduct epithelial spheroids as a model to study sperm-oviduct interactions

**Figure 1:**
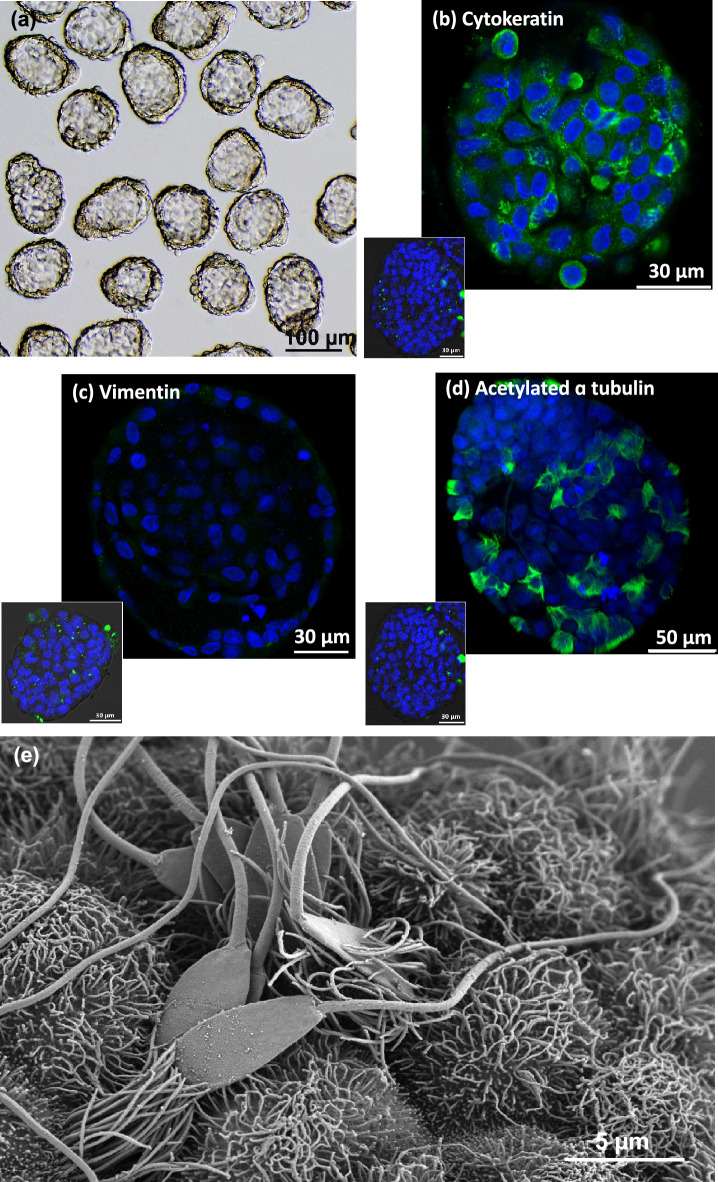
Characterisation of bovine isthmic epithelial spheroids as an in vitro model to study sperm-oviduct interactions. Spheroids that were uniform in shape and size were selected for sperm co-incubation (**a**). The spheroid epithelial cells were stained positively for anti-cytokeratin (**b**, green signal) and negatively for anti-vimentin (**c**) antibodies (nuclei are stained in blue). The sperm-spheroid complex that was immunostained for acetylated alpha-tubulin (in green) and stained with Hoechst (in blue) showed sperm heads bound to cilia (**d**). The sperm-spheroid complex observed by scanning electron microscopy showed sperm heads with an intact acrosome firmly attached to the cilia (**e**). Inserts in (**b**–**d**) show the negative controls incubated with the immunoglobulin isotype of the primary antibody.

Epithelial cell spheroids naturally formed after 2-days of culture of isthmic mucosa fragments collected from post-pubertal cows at the pre-ovulatory stage of their cycle. At day 4, the floating spheroids selected for sperm co-incubation were homogeneous in shape and size (100 µm in diameter) and displayed an epithelium with outward ciliary beating (Fig. 1a; Supplementary Video S1). The immunostaining of the oviduct epithelial spheroids (OES) showed a positive signal for the epithelial marker cytokeratin and a negative signal for the mesenchymal marker vimentin (Fig. 1b, c). On average, 25 ± 2% of cells composing the spheroids were ciliated (n = 100 OES from five replicates; Fig. 1d).

After incubation with OES (see Supplementary Video S2), 85 ± 3% of sperm were found to bind to cilia while the others were bound to non-ciliated cells (n = 100 sperm-OES complexes from five replicates; Fig. 1d, e).

Scanning electron microscopy (SEM) analyses revealed that 93% of the bound sperm were morphologically normal. Among the bound sperm, 93% were attached exclusively with their head and 7% with both their head and midpiece or the flagella (n = 80 spermatozoa from one replicate; Fig. 1e).

**Figure 2:**
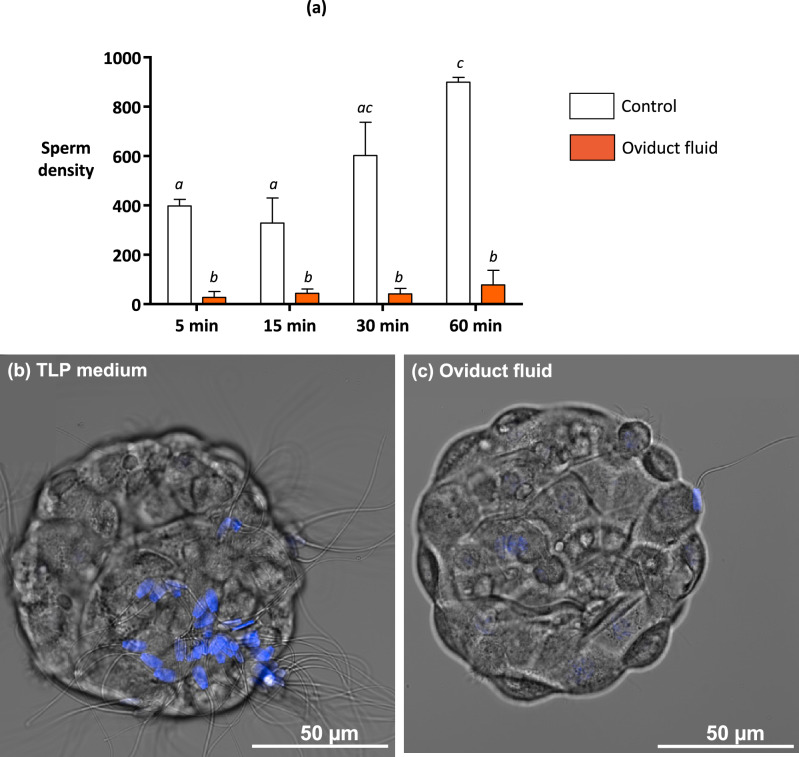
Kinetics of sperm binding to oviduct epithelial spheroids in the Tyrode Lactate Pyruvate (TLP) control medium and in pre-ovulatory oviduct fluid (Experiment 1). (**a**) The spermatozoa and spheroids were incubated in the control TLP medium (white bars) or pre-ovulatory oviduct fluid (OF) at a final protein concentration of 1 mg/mL (orange bars) for 5, 15, 30. and 60 min. The data are provided as the mean ± SEM of bound sperm per mm^2^ (n = 3 replicates). The different letters indicate differences between groups. Confocal microscopy pictures of sperm-spheroid complexes after incubation for 60 min in the TLP medium (left) or pre-ovulatory OF at a 1 mg/mL concentration of proteins (right). The sperm nuclei appear in blue.

Quantitative analyses of sperm binding to OES in the control (Tyrode Lactate Pyruvate or TLP medium) showed that the density of bound spermatozoa increased significantly from 5 to 60 min (Fig. 2). The bound sperm density was repeatable between replicates with different pools of OES (CV of 14%) and reached on average 1416 ± 149 spermatozoa/mm^2^ after 60 min (n = 600 sperm-OES complexes from 30 replicates). The following experiments aimed to study the effect of the OF on sperm binding to OES. The experimental design is summarised in the Supplementary Fig. S1.

### Impact of the oviduct fluid on sperm binding to oviduct epithelial spheroids

Experiment 1 showed that the OF collected at the pre-ovulatory stage of the cycle, *i.e.*, the time at which cows are usually inseminated, significantly decreased sperm binding to OES compared to the control TLP medium (p < 0.05; Fig. 2). In the presence of the OF, the density of bound sperm on OES was 87% lower than in the control as soon as 5 min after sperm addition (p < 0.001) and remained significant throughout the 60-min incubation period (Fig. 2).

The OF at the same protein concentration did not affect any sperm motility parameters nor membrane or acrosome integrity (data on sperm are summarised in the Supplementary Fig. S1). The SEM analyses did not reveal any differences between the OF-treated and control groups in the proportions of spermatozoa bound to the cilia (80% *vs*. 87%), morphologically normal bound spermatozoa (97% *vs*. 93%), those bound exclusively with their head (90% *vs*. 93%), or with their head and midpiece or flagella (10% *vs*. 7%) after 60 min of incubation (n = 60 and 80 spermatozoa from one replicate in the OF-treated and control groups, respectively).

**Figure 3:**
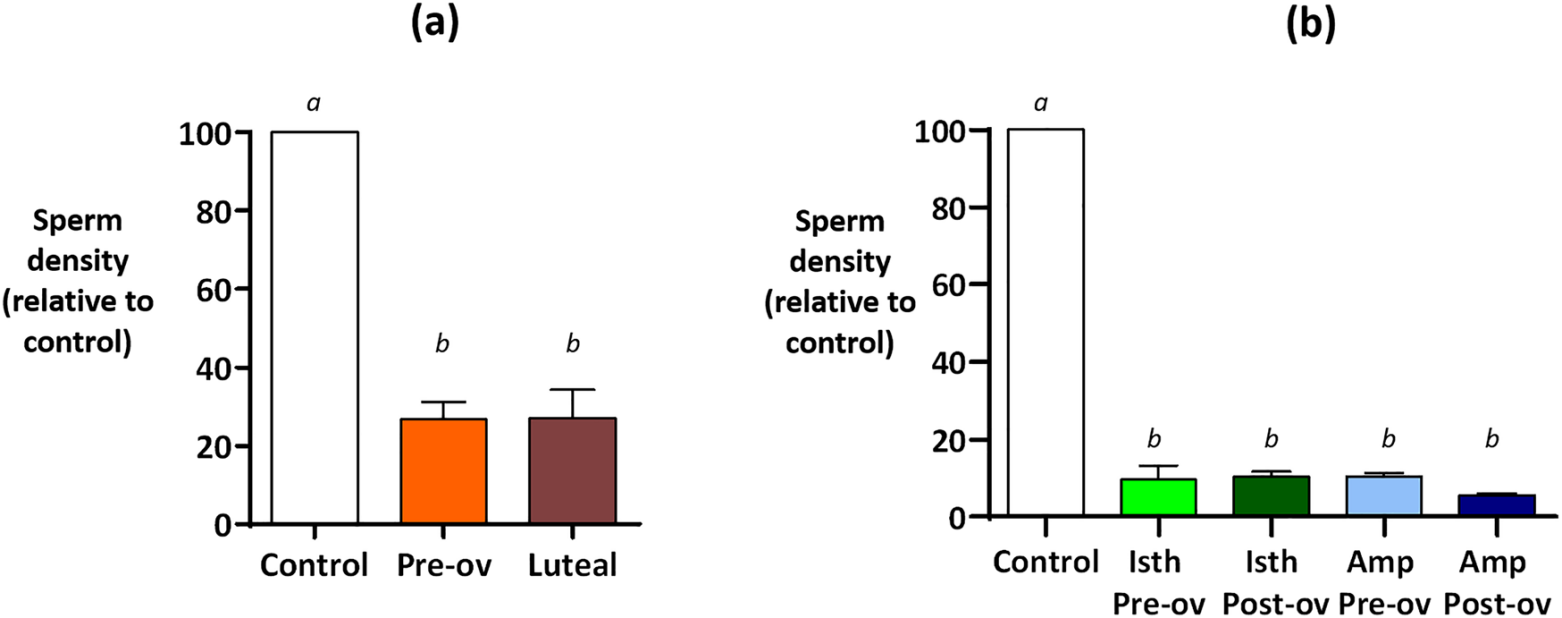
Effect of the stage of the cycle (**a**) and anatomical regions (**b**) from which the oviduct fluid originated on sperm binding to oviduct epithelial spheroids (Experiment 2). The spermatozoa and spheroids were incubated for 60 min in the TLP medium (control) or in the OF collected from whole oviducts at the pre-ovulatory (Pre-ov) or luteal (Luteal) phases of the cycle (**a**), or from the isthmus (Isth) or ampulla (Amp) at pre-ovulatory (Pre-ov) or post-ovulatory (Post-ov) phases of the cycle. All of the OF samples used had a final protein concentration of 1 mg/mL. The data are provided as the mean ± SEM of bound sperm per mm^2^ relative to the control (n = 3 replicates).

Experiment 2 showed that the OF had similar effects on sperm binding to OES regardless of the stage of the cycle (pre-ovulatory *vs*. luteal phase; Fig. 3a) or oviduct anatomical region (isthmus *vs*. ampulla; Fig. 3b) from which the fluid originated. The following experiments aimed to identify which molecules were involved in controlling the effect of OF on sperm binding. In the following, the term OF refers to the fluid collected from whole oviducts at the pre-ovulatory stage of the cycle.

### Impact of macromolecules and secreted proteins on sperm binding to oviduct epithelial spheroids

**Figure 4:**
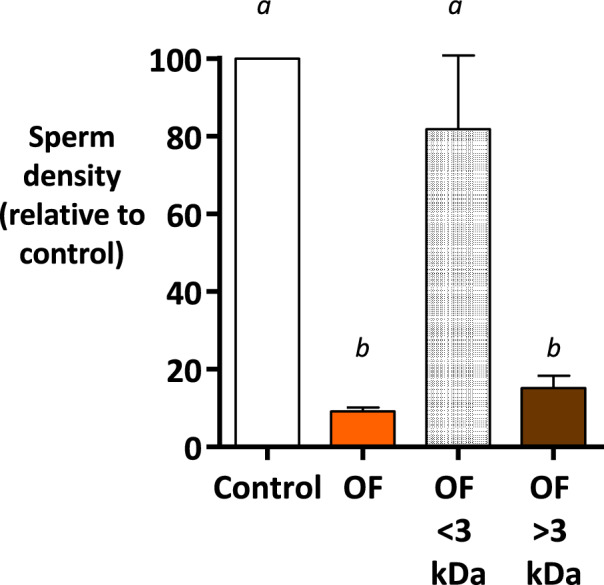
Effect of ultra-filtrated fractions of pre-ovulatory oviduct fluid on sperm binding to oviduct epithelial spheroids (Experiment 3). The spermatozoa and spheroids were incubated for 60 min with pre-ovulatory OF after ultrafiltration with a cut-off of 3 kDa. Native OF at a final protein concentration of 1 mg/mL was included (OF). The TLP medium was used as the control. The data are presented as the mean ± SEM of bound sperm per mm^2^ relative to the control (n = 4 replicates).

Experiment 3 showed that the OF fraction above 3 kDa, but not below 3 kDa, reproduced the effect of the OF on sperm binding, suggesting an involvement of macromolecules (Fig. 4).

**Figure 5:**
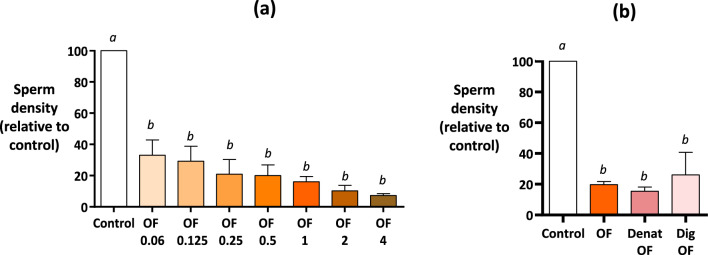
Effect of proteins in the oviduct fluid on sperm binding to oviduct epithelial spheroids (Experiment 4). The spermatozoa and spheroids were incubated for 60 min in the pre-ovulatory OF at final protein concentrations of 0.06, 0.125, 0.25, 0.5, 1, 2, or 4 mg/mL (**a**), or in the OF at a final concentration of 1 mg/mL of proteins (OF) and after protein denaturation by heating (Denat OF) or protein digestion by proteinase K treatment (Dig OF). The TLP medium was used as control (**a**, **b**). The data are provided as the mean ± SEM of the bound sperm per mm^2^ relative to control (n = 4 replicates for each experiment).

Accordingly, Experiment 4 showed that the OF decreased the density of sperm bound on the OES in a protein dose-dependent manner (Fig. 5a). However, the OF in which proteins were denatured by heating (10 min at 100 °C) or enzymatically digested (proteinase K treatment) decreased the bound sperm density at the same extent as the native OF (Fig. 5b).

Sperm motility was negatively affected by OF denaturation but not by the digestion treatment (33.5 and 58.7% of motile spermatozoa, respectively, compared to 65.9% in the control group after 60 min, p < 0.05 for denaturation only; Supplementary Data S1). The results of Experiment 4 indicated that the amino acid chains of soluble proteins and proteins were not involved in determining the effect of the OF on sperm binding to OES.

### Impact of secreted glycosaminoglycans on sperm binding to spheroids

As Experiments 3 evidenced a role of macromolecules in determining the effects of OF, HA (4–8000 kDa) and HS (10–70 kDa)^25^, two glycosaminoglycans that are naturally present in the OF^12^, were tested (Experiment 5).

With concentrations between 10 and 1000 µg/mL, the HA did not have any effect on sperm motility, except for inducing a moderate decrease in curvilinear velocity (VCL) and amplitude lateral head displacement (ALH) at T0. After 60 min, the HS at its highest dose (1000 µg/mL) decreased the proportions of motile and progressive sperm (40.2 and 25.3%, respectively) compared to the control medium (66.0 and 45.8%, respectively, p < 0.05) while at a concentration of 100 µg/mL, it slightly decreased the proportion of progressive sperm without affecting the total motility (see Supplementary Data S1).

**Figure 6:**
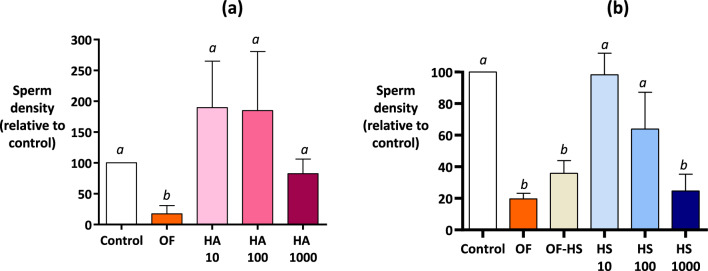
Effect of hyaluronic acid and heparan sulphate on sperm density on bovine oviduct epithelial spheroids (Experiment 5). The spermatozoa and spheroids were incubated for 60 min with hyaluronic acid (HA) at 10, 100, and 1000 µg/mL (**a**), or with heparan sulphate (HS) at 10, 100, and 1000 µg/mL and OF in which the HS was digested with heparinases (OF-HS, **b**). Native OF at a final protein concentration of 1 mg/mL was included (OF) and the TLP medium was used as control in each experiment. The data are presented as the mean ± SEM of bound sperm per mm^2^ relative to the control (n = 4 replicates for each experiment).

At concentrations of 10 and 100 µg/mL, the HA tended to increase the density of sperm bound on OES compared to the control, although this effect was not significant (Fig. 6a). On the contrary, the HS decreased sperm binding on OES in a dose-dependent manner: sperm density on OES was decreased by 36% at 100 µg/mL HS and by 75% at 1000 µg/mL HS relative to the control (*p*-values = 0.1 and < 0.001, respectively; Fig. 6b). In accordance, the OF in which HS was removed by heparinases (OF-HS) had a lower effect on sperm binding than the native OF: compared to control, the sperm density on OES was decreased by 60% with the OF-HS *vs*. by 80% with the native OF (*p*-values < 0.001 and < 0.0001, respectively; Fig. 6b). These results suggest that HS but not HA was partly involved in determining the effect of OF on sperm binding.

## Discussion

Spermatozoa interact with the OF before they bind to the isthmus luminal cells in the so-called sperm reservoir. The present study examined the effect of OF on sperm-oviduct interactions using a newly characterised in vitro model of isthmic epithelial spheroids.

The main findings of this study were that: (1) the OF decreased the number of spermatozoa that bind to OES without affecting sperm motility, membrane integrity, or sperm-cilia interactions; (2) the effect of OF on sperm binding was massive and did not vary according to the cycle stage and oviduct anatomical region that the OF originated from; (3) macromolecules larger than 3 kDa, including HS, were involved in controlling the effect of OF on sperm binding; (4) HA tended to increase sperm binding to OES.

Exploring periconception events in mammals requires the use of in vitro models to overcome the difficulties associated with accessing oviducts by surgery and to avoid the sacrifice of females. According to in vivo observations, spermatozoa bind mostly to ciliated cells in the isthmus^18^, highlighting the importance of keeping epithelial differentiation in vitro. Up to now, most studies on sperm-oviduct interactions have used OEC aggregates or explants a few hours after their collection^2,18,19^ or OEC monolayers reaching confluence after 5–7 days of culture^5,20,24^. One study in pigs mentioned vesicles formed from OEC in a primary culture but without any characterisation of the model: no pictures or immunocytochemistry data were provided^26^. The main limitation of using aggregates or explants is the lack of standardisation of the outward surface and difficulty in assessing the bound sperm density due to numerous folds, whereas the main limitation of OEC monolayers grown on a plastic dish is that the cells rapidly lose their epithelial differentiation and cilia^27^. Here, we used oviduct epithelial spheroids containing well-differentiated epithelial cells and a repeatable proportion of 25% ciliated cells obtained after four days of culture. This proportion of ciliated cells was similar (20–40%) to those previously assessed in sections of the isthmus of post-pubertal cows^28^. Furthermore**,** the spheroids could be selected according to their size (here 100 µm in diameter) and shape (round without folds), providing a standardised model to study sperm-oviduct interactions.

In accordance with our initial hypothesis, the OF decreased sperm binding to OES compared to the control medium. This effect was highly repeatable over more than 30 replicates, decreasing by 80–90% the sperm density per surface unit relative to the control. Surprisingly, this effect was not restricted to the pre-ovulatory period; indeed, the same effect was observed regardless of the stage of the cycle or the anatomical region from which the OF originated. The OF-induced decrease in sperm binding was not due to a decrease in sperm motility or membrane damage during the 60-min incubation period.

This effect was also not due to a difference in binding surface on OES since the spheroids were homogeneous in size and contained similar proportions of ciliated cells (25%). Furthermore, the OF did not display any adverse effects on spheroid cell viability, as evidenced by ethidium homodimer staining (data not shown). Although fewer spermatozoa were bound to the spheroids in the presence of OF, the cilia morphology and sperm-cilia interactions were very similar in both the OF-treated and control groups. The observation of sperm-OES complexes by scanning electron microscopy evidenced close sperm-cilia interactions, with more than 90% of spermatozoa being bound exclusively by their head or by their head and midpiece or flagella. Similar patterns of sperm-cilia interactions have been observed after insemination in cattle^29^ but without any quantitative data. Spermatozoa with an intact acrosome and no morphological abnormalities (for more than 93%) were bound to OES, in line with previous in vivo observations of the bovine oviduct epithelium^18^. In this study, the spermatozoa were used after Percoll gradient centrifugation to remove the commercial extender and obtain homogeneous sperm populations; this likely contributed to the high proportion of spermatozoa with no abnormalities.

The effect of the OF was rapid, the kinetics showing a much lower bound sperm density than in the control group as soon as 5 min after sperm addition. In contrast, sperm binding increased progressively from 5 to 60 min following sperm addition in the control group. Given that both the OES and sperm were in contact with the OF, we cannot exclude the possibility of a combined action of the OF on both the sperm membrane and sperm binding sites at the surface of the spheroids.

Based on our recent proteomic data which identified proteins interacting with bull sperm in the OF, including those previously identified as sperm receptors on oviduct epithelial cells^8^, our hypothesis was that the sperm rapidly interacted with soluble proteins in the OF, preventing further attachment at the surface of OES through a process of binding competition.

The hypothesis of sperm binding competition against OF proteins was supported by the fact that fluid fractions above 3 kDa, but not those below this threshold, reduced the bound sperm density on OES by 90%, similarly to the native OF as compared to the control medium. This result let us to exclude small molecules such as fucose (180 Da), previously identified as an inhibitor of sperm binding to bovine oviduct explants in vitro^30^. This also let us to exclude progesterone (314 Da), previously identified as a sperm releasing factor after binding to OEC in vitro^5,31^. However, when proteins in the OF were digested by proteinase K, the effect on sperm binding was similar to that of the native OF, prompting us to suggest the action of a mechanism independent of the amino acid part of soluble proteins. The proteinase K treatment cut the amino acid chains of proteins but had no effect on the glycan chains linked to these amino acid chains: we cannot exclude that those glycans partly participate in mediating the effect of OF on sperm binding.

Sperm-OEC interactions may be modulated by glycosaminoglycans (GAG). There is evidence from in vitro studies that bull and human sperm bind to HA^14,15,32^. Although existing data are scarce and rather old, HA and HS have previously been reported as the two most abundant GAGs in the OF of post-pubertal cows, accounting for 39 and 26% of the total amounts of GAG, respectively^12^. To our knowledge, there is currently no data available on the amount of HS present in bovine OF. It has been shown that the total amount of HA measured in the OF of dairy cows ranged from 0 to 18.7 µg per day with great variations between individual samples across the cycle and between animals^11^. In this study, the densities of sperm bound to OES in the presence of HA at 10 and 100 µg/mL concentrations tended to be higher than in the control group, without changing sperm motility. Therefore, HA was excluded from the list of OF components responsible for driving the inhibitory effect of the OF on sperm binding. On the contrary, HS, a heavily sulphated GAG, decreased sperm binding to OES in a dose-dependent manner.

Furthermore, the OF deprived of HS was 20% less effective than the native OF in inhibiting sperm binding compared to control, suggesting that HS interacts with sperm and prevents them to bind to OES. In line with our results, HS but not HA has previously been shown to bind with a high affinity to purified BSPs^16^. The BSP-1 (also called PDC-109 or BSP-A1/A2), BSP-3 (or BSP-A3), and BSP-5 (or BSP-30KDa) are seminal plasma-derived proteins which coat the bull sperm surface at ejaculation and play a key role in sperm binding to OEC^33,34^. The proteins of the BSP family have several interaction partners, including sulphated GAGs and annexins, which may explain their role in regulating sperm binding to the oviduct reservoir^35^. It has been hypothesised that GAGs close to heparin are released from the follicular fluid into the oviduct at the time of ovulation, allowing for sperm detachment from the reservoir through interactions with both BSPs and annexins for subsequent fertilisation^35^. According to our results, this scenario is unlikely: the HS in the OF collected before ovulation already had an inhibitory effect on sperm binding, and the effect of the OF did not differ depending to the cycle stage. Therefore, it is likely that instead of triggering sperm detachment, the HS in the oviduct lumen restrains sperm adhesion to the reservoir during the whole peri-ovulatory period.

One limitation of this study was the lack of information on animals used for the OF and cell collection since the samples were collected at a slaughterhouse. However, the effect of the OF on sperm binding was highly reproducible over three years of experiments, regardless of the collection date and animal breed. In addition, the same pool of semen was used in all experiments in order to focus on the female factors regulating sperm-oviduct interactions, but the effect of the OF may vary from one bull to another. Another limitation is that, due to the collection process which employed flushing, the OF was diluted. It could be hypothesised that the inhibitory effect of the OF on sperm binding is even stronger in vivo and contribute to the selection of a sperm subpopulation capable of binding to the isthmic reservoir and be capacitated prior to ovulation. Although the apical cell surface structure of the OES and sperm binding to ciliated cells looked comparable to previous observations in vivo^18,28^, the sperm density and binding modulation by the OF may differ from what is happening at the isthmus in situ.

In conclusion, this study proposed a new in vitro model of an oviduct sperm reservoir, with the isthmic epithelial spheroids containing 25% of ciliated cells. The model was easily reproducible and could be standardised for further studies on sperm-oviduct interactions in different fluidic environments. The present study evidenced for the first time that the macromolecules present in the OF, including HS, rapidly decreased the number of spermatozoa that bind to isthmic epithelial cells. Further work is required to explore the roles of other OF macromolecules on sperm-oviduct interactions and the functional roles of sperm selective binding in the isthmus on subsequent fertilisation.

## Methods

### Media and reagents

The chemicals used for the media were obtained from Sigma-Merck (Missouri, United States) and ThermoFisher Scientific (Massachusetts, USA). The HEPES (4-(2-hydroxyethyl)-1-piperazine ethane sulfonic acid)-buffered Tyrode’s lactate pyruvate medium (TLP medium) contained 100 mM NaCl, 3.1 mM KCl, 0.3 mM NaH_2_PO_4_, 2.1 mM CaCl_2_, 0.4 mM MgCl_2_, 1 mM sodium pyruvate, 27.4 mM sodium lactate, 10 mM HEPES, and 1 mg/mL polyvinyl alcohol (PVA)^21,36^. PVA was used instead of albumin to avoid sperm capacitation^37^. The pH of the TLP medium was adjusted to 7.4 with NaOH 1 M and the osmolarity to 300 mOsm with NaCl. The TLP medium was stored at 4 °C for a maximum of 2 weeks. The M199 culture medium (Medium 199, Earle’s salts; 31150-022) was supplemented with 2.5% HEPES (15630-080, ThermoFisher Scientific, Massachusetts, United States), 0.5% of gentamycin (G1272, Sigma, Missouri, United States), and 10% heat-treated fetal calf serum (A5256701, ThermoFisher Scientific, Massachusetts, United States).

The media were passed through sterile 0.22-µm filters (SLGV013SL) before use. The TLP medium was placed at 38.8 °C for a minimum of 1 h before use for sperm incubation or at 4 °C before use for oviduct flushing. The M199 medium was placed at 38.8 °C under humidified atmosphere containing 20% O_2_ and 5% CO_2_ for a minimum of 1 h before its use for the cell culture. Unless otherwise specified, reagents were obtained from Sigma-Merck.

### Culture of isthmic mucosa fragments and selection of oviduct epithelial spheroids (OES)

Bovine oviducts and ovaries were collected from a local slaughterhouse (Vendôme, 45 min from the laboratory) and transported on ice to avoid post-mortem modifications. Pairs of oviducts from two cows at a pre-ovulatory stage of their cycle (one follicle of 11–20 mm in diameter and a *corpus albicans*, *i.e.*, corresponding to approximately days 19–21 of the estrous cycle) were isolated from surrounding vessels and tissues and cut at the utero-tubal and ampullary junctions to isolate the isthmus (around 5 cm long)^38^. The isthmic sections were rapidly washed in 70% ethanol, rinsed in NaCl 0.9%, then both extremities (around 5 mm) were cut with a sterile scalpel to avoid any contamination. The isthmus mucosa was then expelled with forceps in 10 mL of a M199 culture medium, vortexed for 1 min, then incubated at 38.8 °C for a minimum of 10 min for cell sedimentation. Following the elimination of the supernatant, the pellet was resuspended in 10 mL of the culture medium and the vortex-sedimentation process was repeated. Finally, the pellet was diluted ten times in the culture medium and 50 µL of the resulting mixture was added to 450 µL of the M199 medium. The isthmic mucosa fragments were cultured for four days in four-well culture plates (176740, ThermoFisher Scientific, Massachusetts, USA) at 38.8 °C in a humidified atmosphere containing 20% O_2_ and 5% CO_2_. On day 4, a cavity was formed within the mucosa fragments, forming spheroids of various sizes and shapes, with the apical side of the epithelial cells oriented outward as determined by the presence of motile cilia.

Spheroids of approximately 100 µm in diameter, homogeneous in form, with vigorous cilia beating, and no surface folding were selected for the sperm binding studies (Fig. 1a; Supplementary Video S1).

### Cell viability and immunostaining of oviduct epithelial spheroids (OES)

The cell viability of the OES was assessed after four days in a M199 medium and up to 4 h in the TLP and OF media at 0.06 and 4 mg/mL of proteins (in which their incubation with spermatozoa was performed) after immunostaining with ethidium homodimer-1 (L3224, Invitrogen, Massachusetts, USA). The OES were incubated in 500 µL of the culture medium and 1 µL of ethidium homodimer-1 and Hoechst (B2261) for 45 min at 38.8 °C in a humidified atmosphere containing 20% O_2_ and 5% CO_2_, before being directly observed by confocal microscopy (Zeiss LSM 700, Carl Zeiss, Oberkochen, Germany).

To further characterise the OES, immunostaining for pan-cytokeratin (epithelial cell marker), vimentin (stromal cell marker), and acetylated alpha-tubulin (marker of ciliated cells) was performed as previously described^39,40^. Briefly, the OES were fixed (4% paraformaldehyde in PBS supplemented with 0.5% of Triton X-100 and 1% of bovine serum albumin (PBS-BSA), 1 h at 37 °C), washed three times in PBS-BSA, and incubated in a blocking solution (10% goat serum in PBS-BSA, 30 min, room temperature). The OES were then incubated overnight at room temperature with the primary antibody (anti-cytokeratin, C2931; anti-vimentin, V6630; anti-acetylated tubulin, T7451; final concentrations of 30, 100, and 2 µg/mL, respectively). Immunostaining for acetylated alpha-tubulin (T7451; final concentration of 2 µg/mL) was performed on the sperm-OES complexes to assess the proportions of sperm bound to the ciliated cells. The control OES were incubated with IgG1 (M9269) at the same concentration as the primary antibody. After washing in PBS-BSA, the OES were incubated with the secondary antibody coupled with Alexa Fluor 488 (A11001, Invitrogen, Massachusetts, USA; final concentration at 2 µg/mL) for 2 h at room temperature under agitation.

After washing in PBS-BSA, the OES or sperm-OES complexes were stained with Hoechst (B2261, 10 µg/mL) and observed under confocal microscopy. For each antibody, a total of 100 OES from five different replicates were evaluated.

### Preparation of spermatozoa, assessment of sperm motility, and membrane integrity

A pool of semen from the same ejaculates of two Normande and one Holstein bulls (Union Evolution, Noyal-sur-Vilaine, France) and previously frozen in Optidyl extender (IMV technologies, L’Aigle, France) were used for all experiments. Semen samples were thawed 3 min in a water bath at 37 °C then washed on a 45–90% Percoll gradient by centrifugation (700 × *g*, 20 min, 25 °C). An aliquot of the sperm pellet was used to assess the sperm concentration in a Thoma cell counting chamber. The sperm were then washed in 1 mL of the TLP medium for 10 min at 300 × *g* at 25 °C prior to their incubation with spheroids. Just before and at the end of each incubation, the sperm motility was assessed using computer-assisted sperm analysis (CASA, IVOS II, IMV technologies). The proportions of motile spermatozoa before and after incubation typically exceeded 70 and 60%, respectively (Supplementary Data S1). In some experiments, aliquots of spermatozoa were analysed by flow cytometry (MACSQuant Analyser 10, Miltenyi Biotec, Germany) following their incubation for 10 min at room temperature in 200 µL of TLP with 7.5 µM propidium iodide (PI, Sigma 81845) and 1 µM peanut agglutinin fluorescein (PNA, Vector FL-1071) to assess the sperm membrane integrity and acrosome reaction, respectively.

### Sperm addition to spheroids and quantification of bound sperm density

Two hours before the sperm addition, the OES were briefly washed in a M199 medium then transferred into 500 µL of the TLP medium at 38.8 °C (with no CO_2_ to avoid the formation of bicarbonate).

During the Percoll centrifugation (30 min prior to sperm addition), groups of 20 OES in 10 µL of TLP medium were transferred to 50 µL of the medium (TLP or treatment) in a sterile 96-well culture plate at 38.8 °C. Sperm were then added to 5 µL of TLP so that the final volume of sperm-OES incubation was 65 µL. Preliminary data using increasing concentrations of spermatozoa added to 20 OES in the TLP medium showed that the density of bound sperm reached a plateau at 1 million of spermatozoa per mL. Therefore, the final sperm concentration added to a group of 20 OES was 1 million spermatozoa per mL in a final volume of 65 µL (see Supplementary Video S2). After the incubation, the sperm-OES complexes were gently washed three times in 500 µL of TLP medium at 38.8 °C using a glass pipette (400 µm in diameter) in order to eliminate slightly attached spermatozoa, then immediately fixed in 2.5% glutaraldehyde diluted in 0.1 M sodium cacodylate (27746.180, Prolabo, France) for 1 h at 37 °C. After three washes in PBS-BSA, the sperm-OES complexes were kept at 4 °C overnight until further analyses. The preliminary experiments showed that this procedure did not induce sperm detachment from the OES. The nuclei of both the sperm and OES were stained with Hoechst (B2261; 10 µg/mL) for 30 min under gentle agitation at room temperature. After two washes in PBS-BSA, the OES were placed on a Superfrost slide (J1800AMNZ, Epredia, Breda, Netherlands), mounted under a coverslip, and analyzed by confocal microscopy (Zeiss LSM 700, Oberkochen, Germany). In order to visualise all sperm heads, both sides of the OES were observed under a 405 nm excitation wavelength and pictures were saved. The total number of bound spermatozoa per OES and areas of OES were quantified using the ImageJ software (version 1.53t). The bound sperm density was calculated as the total number of sperm bound on the two sides of the spheroids divided by the total area of spheroids.

### Collection and preparation of oviduct fluid (OF) samples

Bovine OF were collected from adult cyclic cows at a local slaughterhouse. Pairs of oviducts and ovaries were immediately placed on ice, transported to the laboratory, and classified according to their ovarian morphology as previously described^38^. Oviducts which were ipsilateral and contralateral to ovulation at either the pre-ovulatory, post-ovulatory (presence of an early corpus luteum consisting of red, loosely organised tissue of less than 1 cm in diameter, approximately days 1–4 of the cycle), or luteal stage of the cycle (presence of a well-formed brown or orange *corpus luteum* of more than 1 cm in diameter) were used. The ovaries, surrounding tissues, and infundibulum were removed before flushing with the TLP medium at 4 °C. For most experiments, the whole oviducts were flushed with 500 µL of TLP. In some cases, the oviducts were cut at the ampullary-isthmus junction and each section (isthmus and ampulla) was flushed separately with 200 µL of TLP. The cells and cellular debris were discarded through two successive centrifugations (2000 × *g* for 10 min at 4 °C then 12,000 × *g* for 10 min at 4 °C). An OF aliquot was used for protein concentration using the Uptima BC Assay kit (Interchim, Montluçon, France). The protein concentrations of the OF at the collection time ranged from 1 to 4 mg/mL. The OF samples used for incubation with OES and sperm consisted of pools of OF collected from ipsi- and contralateral oviducts from two cows at the same stage of their estrous cycle and these were stored at − 80 °C until use (one freezing–thawing cycle before use). The final protein concentration was adjusted at the time of thawing.

To obtain the OF filtration fractions, 500 µL of pre-ovulatory OF at a final protein concentration of 1 mg/mL were centrifuged on a Vivaspin® ultrafiltration unit (3-kDa cut-off; VS0191, Sartorius, Göttingen, Germany) at 12,000 × *g* for 1 h at 4 °C. The fractions at the top (above 3 kDa) and at bottom (below 3 kDa) of the unit were collected, assayed for protein concentration, and stored at − 20 °C until use. Sodium dodecyl sulfate–polyacrylamide gel electrophoresis (SDS-PAGE) was conducted to check the efficiency of the ultrafiltration.

For protein denaturation, pre-ovulatory OF with a 1 mg/mL concentration of proteins was treated for 10 min at 100 °C, then kept at room temperature for 20 min before its use. For protein digestion, pre-ovulatory OF with a protein concentration of 1 mg/mL was incubated in a TLP medium supplemented with 200 µg/mL of proteinase K (A3830, AppliChem, Germany) overnight at 56 °C.

The proteinase K was discarded from the OF through ultrafiltration (Vivaspin® 50 kDa cut-off, VS0132, Sartorius, Göttingen, Germany) for 10 min at 12,000 × *g*. A Bradford protein assay (Interchim, Montluçon, France) and SDS-PAGE were performed to assess the efficiency of the digestion and elimination of proteinase K. The proteinase K-free digested OF fraction below the 50 kDa cut-off was stored at − 20 °C before use.

In order to eliminate the HS from the OF, pre-ovulatory OF with 1 mg/mL of proteins was incubated in a TLP medium supplemented with a mixture of heparinases I, II, and III (H3917, H6512, v/v) with a final concentration of 0.1 UI/mL for 5 h at 37 °C.

The heparinases were discarded from the OF via ultrafiltration (Vivaspin® 50 kDa cut-off, VS0132, Sartorius, Göttingen, Germany) for 10 min at 12,000 × *g* at 4 °C. SDS-PAGE was performed to assess the elimination of heparinases from the OF. The treated samples were stored at -20 °C before use.

### Observation of sperm-cilia interactions by scanning electron microscopy

Sperm-OES complexes were immersed in a fixative solution (2,5% glutaraldehyde in 0.1 M sodium cacodylate buffer, pH 7.4) and stored at 4 °C until processing. The fixative was removed, and samples were rinsed in the sodium cacodylate solution (pH 7.4). They were deposited on sterile cover-glasses discs (Marienfeld, VWR, France). The samples underwent progressive dehydration by soaking in a graded series of ethanol (50 to 100%) before critical-point drying under CO_2_.

Samples were mounted on aluminum SEM sample stubs (15 mm diameter × 6 mm M4, Micro to Nano, Haarlem, Netherlands) with carbon adhesive discs (Agar Scientific, Oxford Instruments SAS, Gometz-la-Ville, France) and sputter coated with Gold–Palladium (Polaron SC7640, Milexia, Verrières-le-buisson, France) for 220 s at 10 mA. Samples were visualized by field emission gun scanning electron microscopy (SEM FEG). They were viewed as secondary electron (2 kV) with a Hitachi S4500 instrument (Milexia, Verrières-le-buisson, France). Samples preparation and scanning Electron Microscopy analyses were performed at the Microscopy Platform of the INRAE PACA center, Avignon, France.

### Experimental design

The general design of the experiments is presented in the Supplementary Fig. S1. For all experiments, sperm motility was assessed by computer-assisted sperm analysis (CASA) at 0 and 60 min of incubation with native OF and the TLP medium as controls.

**Experiment 1** evaluated the kinetics of sperm binding to OES under control conditions and with OF. The sperm and OES were incubated for 5, 15, 30, and 60 min in the TLP medium or in the presence of pre-ovulatory OF at a final protein concentration of 1 mg/mL.

**Experiment 2** evaluated the effect of the stage of the cycle (pre-ovulatory *vs*. luteal phase of cycle) and anatomical region of the oviduct (ampulla *vs*. isthmus) from which the OF originated on sperm binding. For each experiment, the sperm and OES were incubated for 60 min in the presence of OF with a final protein concentration of 1 mg/mL.

Preliminary experiments showed that more than 99% cells in the OES were viable (showed a negative signal for ethidium homodimer) after 60 min in TLP and OF. The TLP medium was used as the control.

**Experiment 3** evaluated the effect of ultra-filtrated fractions OF on sperm binding. The sperm and OES were incubated for 60 min in > and < 3 kDa fractions of pre-ovulatory OF. The TLP medium was used as the control.

**Experiment 4** evaluated the effect of the OF proteins on sperm binding. The sperm and spheroids were incubated for 60 min with pre-ovulatory OF with 0.06, 0.125, 0.25, 0.5, 1, 2, or 4 mg/mL concentrations of proteins. The sperm and OES were incubated for 60 min in pre-ovulatory OF in which the proteins were denatured by heating or digested by proteinase K. The TLP medium and native OF were used as the controls.

**Experiment 5** evaluated the effect of HA and HS on sperm binding. The sperm and OES were incubated for 60 min in pre-ovulatory OF treated with heparinases I, II, and III (OF-HS). In addition, purified HS (H7640) and HA (53747) diluted to 10, 100, and 1000 µg/mL in the TLP medium were tested. These concentrations were chosen based on a preliminary measurement of sulfated GAG in one pool of OF (60 µg/mL) using the Blyscan kit (B1000, Biocolor Ltd, Carrickfergus, United Kingdom). The TLP medium and native OF were used as the controls.

Each experiment was repeated at least three times with OES and OF samples from different cows.

### Statistical analysis

Unless specified otherwise, the data are presented as the mean ± SEM of bound sperm density per mm^2^ relative to the control group in the TLP medium. The statistical analysis was performed in the GraphPad Prism software (version 8.1.1). The bound sperm densities and motility parameters for different conditions were compared using ANOVA and Tuckey’s post-tests for pair-wise comparisons. The proportions of spermatozoa observed by SEM were compared with Chi-square tests. A *p*-value < 0.05 was considered significant.

## Supplementary Information

Supplementary Figure S1.

Supplementary Legends.

Supplementary Table S1.

Supplementary Video 1.

Supplementary Video 2.
