# Supplementary material for: Sperm-fluid-cell interplays in the bovine oviduct: glycosaminoglycans modulate sperm binding to the isthmic reservoir
Source: Sci Rep. 2023 Jun 26;13:10311. doi: 10.1038/s41598-023-37469-3 (PMC10293210; doi:10.1038/s41598-023-37469-3)
Supplement: Supplementary file 1 — Supplementary Figure S1. [file 41598_2023_37469_MOESM1_ESM.pdf]

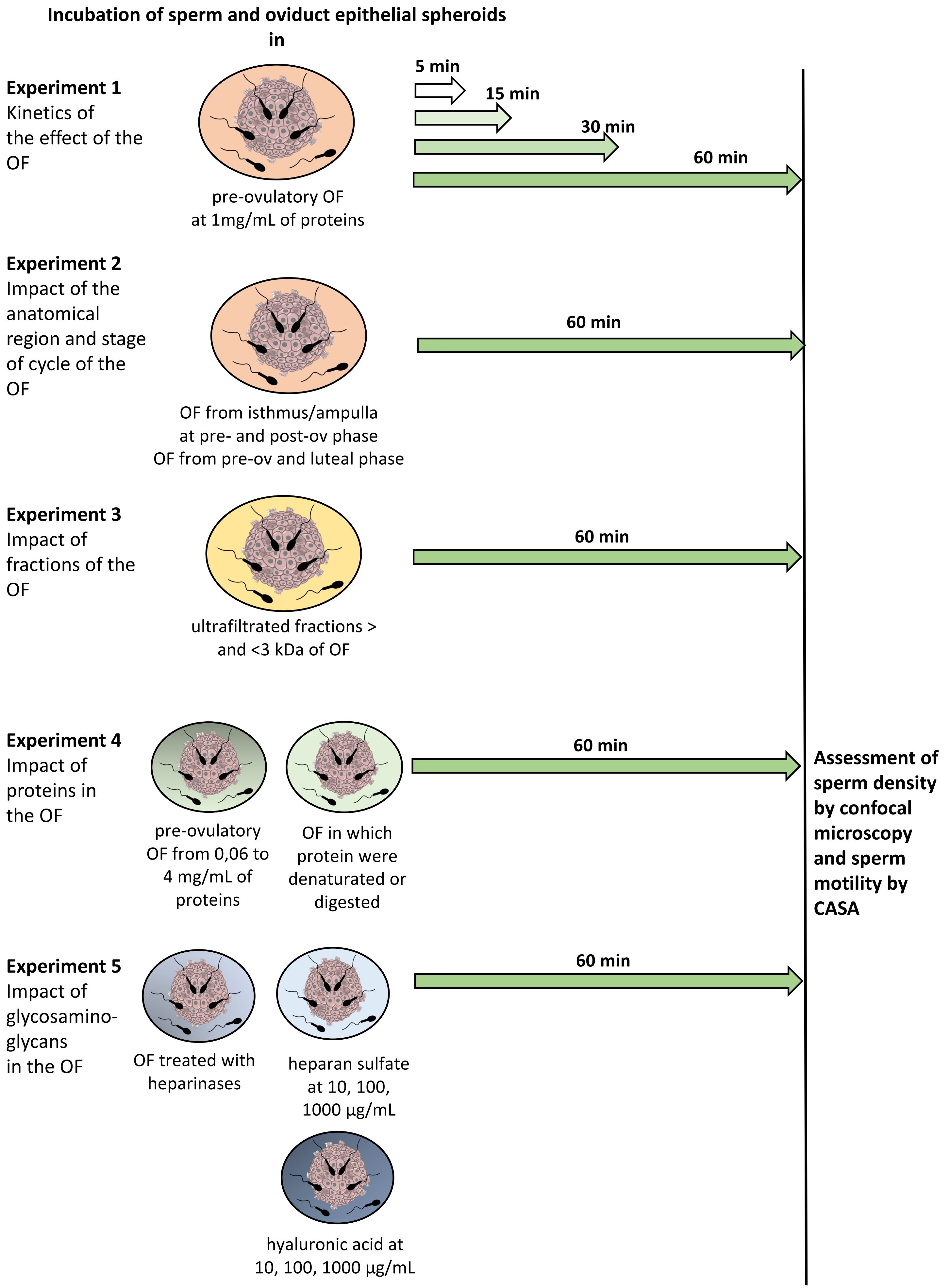
Incubation of sperm and oviduct epithelial spheroids in
Experiment 1
Kinetics of the effect of the OF
pre-ovulatory OF at 1mg/mL of proteins
5 min
15 min
30 min
60 min
Experiment 2
Impact of the anatomical region and stage of cycle of the OF
OF from isthmus/ampulla at pre- and post-ov phase
OF from pre-ov and luteal phase
60 min
Experiment 3
Impact of fractions of the OF
ultrafiltrated fractions > and <3 kDa of OF
60 min
Experiment 4
Impact of proteins in the OF
pre-ovulatory OF from 0,06 to 4 mg/mL of proteins
OF in which protein were denaturated or digested
60 min
Experiment 5
Impact of glycosamino-glycans in the OF
OF treated with heparinases
heparan sulfate at 10, 100, 1000 µg/mL
hyaluronic acid at 10, 100, 1000 µg/mL
60 min
Assessment of sperm density by confocal microscopy and sperm motility by CASA
