# Supplementary material for: Sperm-fluid-cell interplays in the bovine oviduct: glycosaminoglycans modulate sperm binding to the isthmic reservoir
Source: Sci Rep. 2023 Jun 26;13:10311. doi: 10.1038/s41598-023-37469-3 (PMC10293210; doi:10.1038/s41598-023-37469-3)
Supplement: Supplementary file 2 — Supplementary Legends. [file 41598_2023_37469_MOESM2_ESM.docx]

**Supplementary information**

**Sperm-fluid-cell interplays in the bovine oviduct: glycosaminoglycans modulate sperm binding to the isthmic reservoir**

Coline Mahé^1,*^, Thanya Pranomphon^1,2^, Karine Reynaud^1^, Ludivine Laffont^1^, Thierry Meylheuc^3^, Jennifer Schoen^4,^, Pascal Mermillod^1^, Marie Saint-Dizier^1,5^

^1^ CNRS, IFCE, INRAE, Université de Tours, PRC, 37380, Nouzilly, France

^2^ Embryo Technology and Stem Cell Research Center, School of Biotechnology,

Suranaree University of Technology, Nakhon Ratchasima, Thailand

^3^ INRAE, Pathologie Végétale, F-84143 Avignon, France

^4^ Department of Reproduction Biology, Leibniz Institute for Zoo and Wildlife Research (IZW), Berlin, Germany

^5^ Tours University, Faculty of Sciences and Techniques, Tours, France

*Corresponding author**:** coline.mahe@inrae.fr or marie.saint-dizier@univ-tours.fr

**Supplementary Figure 1. Experimental design for sperm-oviduct epithelial spheroids incubation.**

**Supplementary Table S1. Sperm motility, membrane and acrosome integrity assessment during 1 h in control and in each treatment for sperm-spheroids incubation.**

**Supplementary Video S1. Bovine oviduct epithelial spheroids in Tyrode Lactate Pyruvate (TLP) medium after selection according to their shape and size (100 µm-diameter). Spheroids are moving due to ciliary beating on the external surface.**

**Supplementary Video S2. Bovine oviduct epithelial spheroids after sperm addition.**
